# Patient-perceived barriers to early initiation of prenatal care at a large, urban federally qualified health center: a mixed-methods study

**DOI:** 10.1186/s12884-024-06630-9

**Published:** 2024-06-21

**Authors:** Valerie N. Holt, Elan Pelegrí, Mary Hardy, Lindsey Buchin, Isaac Dapkins, Meleen Chuang

**Affiliations:** 1 A.T. Still University – School of Osteopathic Medicine in Arizona, 5850 E. Still Circle, Mesa, AZ 85206 USA; 2 Family Health Centers (FHCs) at NYU Langone, 5610 2nd Avenue, Brooklyn, NY 11220 USA

**Keywords:** Initiation of care, First trimester prenatal care, Barriers to prenatal care, Federally qualified health center, Process improvement, PDSA

## Abstract

**Background:**

Early initiation of prenatal care is widely accepted to improve the health outcomes of pregnancy for both mothers and their infants. Identification of the various barriers to entry into care that patients experience may inform and improve health care provision and, in turn, improve the patient’s ability to receive necessary care.

**Aim:**

This study implements a mixed-methods approach to establish methods and procedures for identifying barriers to early entry to prenatal care in a medically-vulnerable patient population and areas for future quality improvement initiatives.

**Methods:**

An initial chart review was conducted on obstetrics patients that initiated prenatal care after their first trimester at a large federally qualified health center in Brooklyn, NY, to determine patient-specified reasons for delay. A thematic analysis of these data was implemented in combination with both parametric and non-parametric analyses to characterize the population of interest, and to identify the primary determinants of delayed entry.

**Results:**

The age of patients in the population of interest (*n* = 169) was bimodal, with a range of 15 – 43 years and a mean of 28 years. The mean gestational age of entry into prenatal care was 19 weeks. The chart review revealed that 8% recently moved to Brooklyn from outside of NYC or the USA. Nine percent had difficulty scheduling an initial prenatal visit within their first trimester. Teenage pregnancy accounted for 7%. Provider challenges with documentation (21%) were noted. The most common themes identified (*n* = 155) were the patient being in transition (21%), the pregnancy being unplanned (17%), and issues with linkage to care (15%), including no shows or patient cancellations. Patients who were late to prenatal care also differed from their peers dramatically, as they were more likely to be Spanish-speaking, to be young, and to experience a relatively long delay between pregnancy confirmation and entry into care. Moreover, the greatest determinant of delayed entry into care was patient age.

**Conclusion:**

Our study provides a process for other like clinics to identify patients who are at risk for delayed entry to prenatal care and highlight common barriers to entry. Future initiatives include the introduction of a smart data element to document reasons for delay and use of community health workers for dedicated outreach after no show appointments or patient cancellations.

**Supplementary Information:**

The online version contains supplementary material available at 10.1186/s12884-024-06630-9.

## Introduction

### Maternal and child health in New York City

Worldwide, the leading cause of mortality and morbidity in women of reproductive age are complications during pregnancy and childbirth. The World Health Organization (WHO) defines maternal mortality as “the death of a woman while pregnant or within 42 days of termination of pregnancy, irrespective of the duration and site of the pregnancy, from any cause related to or aggravated by the pregnancy or its management but not from unintentional or incidental causes” [1].

Despite its robust healthcare infrastructure, the United States continues to lag behind other developed countries in regard to maternal and child health outcomes. [2]. In 2021, the maternal mortality rate in the US was 32.9 deaths per 100,000 live births, an increase from 23.8 deaths per 100,00 live births in 2020, and 20.1 in 2019 [3]. Black women are disproportionately impacted, with a maternal mortality rate twice that of White women nationwide (37.1 versus 14.7 deaths per 100,000 live births) [4]. In New York City, the maternal mortality was 19.1 per 100,000 between 2018 – 2020, with the highest rates in the Bronx (23.1) and Kings County (21.9) [5]. At the city-level, the racial disparity is further increased, with Black women four times as likely to die during pregnancy or in the year after than compared to White women [6].

Closely linked to maternal health is the health of the infant. The average infant mortality rate in the United States is 5.4 deaths per 1,000 live births [7]. In New York City, this rate decreases to 3.9 deaths per 1,000 live births. In certain neighborhoods, like Greenwich Village and Soho in Manhattan and Bay Ridge in Brooklyn, the infant mortality rate is as low as 0.9 per 1,000. However, the East Flatbush neighborhood in Brooklyn has an infant mortality rate of 6.7 per 1,000, and is one of three neighborhoods in NYC with the highest IMR [8]. These disparities reflect the composition of these neighborhoods. East Flatbush is 50% foreign-born, 78.1% Black, and has a median household income of $64,520 [9]. In contrast, Greenwich Village has a population that is 22.7% foreign born, 66.4% white, and has a median household income of $151,240 [10].

### Early entry to prenatal care

Timely entry into prenatal care, paired with regular prenatal care visits throughout the pregnancy, has been shown to significantly improve health outcomes for both mothers and their infants [11]. Prenatal care ensures the successful delivery of a healthy newborn while decreasing maternal risk by supporting both the early detection and mitigation of maternal and fetal complications in pregnancy. Components of prenatal care include timely, accurate estimation of gestation age; identification of pregnancies at greater risk of maternal and fetal morbidity and mortality, such as women with gestational diabetes or hypertensive disease in pregnancy; continuous evaluation of mother and fetus; and education and support for the parents [12]. Data suggest that no prenatal care or few prenatal visits are associated with maternal death and severe maternal morbidity. Numerous studies illustrate an association between fewer prenatal visits and worse pregnancy outcomes such as low birthweight, preterm birth, and infant mortality [13].

The length of pregnancy is divided into three trimesters. First trimester is from the last menstrual period to 13 weeks and 6 days; second trimester is from 14 to 27 weeks and 6 days; and third trimester lasts from 28 to 40 weeks and 6 days [14, 15]. Prenatal care should be started in the first trimester, ideally before 10 weeks of gestation, as there are prenatal screening and diagnostic tests that can be conducted at 10 – 11 weeks. Early initiation of care is also helpful to determine gestational age and early baseline maternal measurements including weight, blood pressure, and laboratory testing; and to offer early social service support and intervention, as needed [12].

Despite the importance of early prenatal care, medically vulnerable populations are less likely to initiate prenatal care during the first trimester [16]. In a study of urban, inner-city women in Canada, barriers to adequate prenatal care could be understood through the socio-ecological model of health care utilization, falling into individual, interpersonal, and health system challenges. Personal challenges included child care, transportation, addiction, and lack of support. Health provision and system-level challenges included provider qualities (lack of time or negative behavior), provider shortages, and limited availability for appointments [17].

Data from the 2017 Uniform Data System revealed that, among federally qualified health centers (FQHCs), only 57.4% met the Healthy People 2020 baseline of 77.1% of female patients receiving prenatal care in their first trimester. 37.9% met the Healthy People 2020 target of 84.8% of female patients receiving timely prenatal care. Health centers were less likely to meet this target if they were located outside of New England, were located in a rural area, or had a relatively large population of prenatal patients below 15 years old. Additional factors negatively associated with achieving the target included simply servicing a larger prenatal population, providing prenatal care to women with HIV, or having more uninsured patients or patients dually eligible for Medicaid and Medicare [18].

### Family Health Centers at NYU Langone – study aim

Given the vulnerable patient populations served by FQHCs, continued efforts should be focused identifying care gaps from the patient perspective and implementing initiatives to reduce them. As such, this study aims to identify the factors that contribute to the delayed initiation of prenatal care, using Family Health Centers at NYU Langone (FHC), an FQHC in Brooklyn, New York, as a case study. With a mixed methods approach, we used a socio-ecologic framework to qualitatively identify factors impacting early initiation of prenatal care and performed a quantitative analysis to identify patient characteristics associated with being at risk for delayed entry to care.

Family Health Centers serves 100,000 unique patients annually, with 600,000 patient visits budgeted for each year, in Brooklyn, NY. The FQHC’s primary services areas including Sunset Park, Flatbush, Boro Park, Bay Ridge, and Kensington. 43% of these patients are prefer a language other than English. Almost two-thirds of patients are enrolled in Medicaid or a Medicaid managed program. Over 60% of patients are 200% below the Federal Poverty Line, which in 2021, was defined as a single person making less than $25,760 or a family of four making less than $53,000 annually. Family Health Centers provides women’s health services, gynecologic care, and obstetric (OB) care across seven clinic locations in Brooklyn, NY. In 2021, FHC provided care to 1,580 prenatal patients across all locations, of whom 1,140 delivered. Of all the OB patients seen, 81.16% sought access to prenatal care during their first trimester, a slight decrease from 82.5% in 2020, and 86.4% in 2019 [19]. Though FHC exceeds the Healthy People 2030 goal that 80.5% of pregnant patients receive prenatal care during their first trimester, [20] efforts to ensure this care gap decreases and to target the most vulnerable patients remain a priority. The composition of its patient population, in combination with its relatively robust data infrastructure, make FHC an ideal health system to evaluate. Our methods and findings can be generalized to other clinics that serve similar diverse populations to support universal efforts to ensure high quality care of birthing parents and infants.

**Fig. 1:**
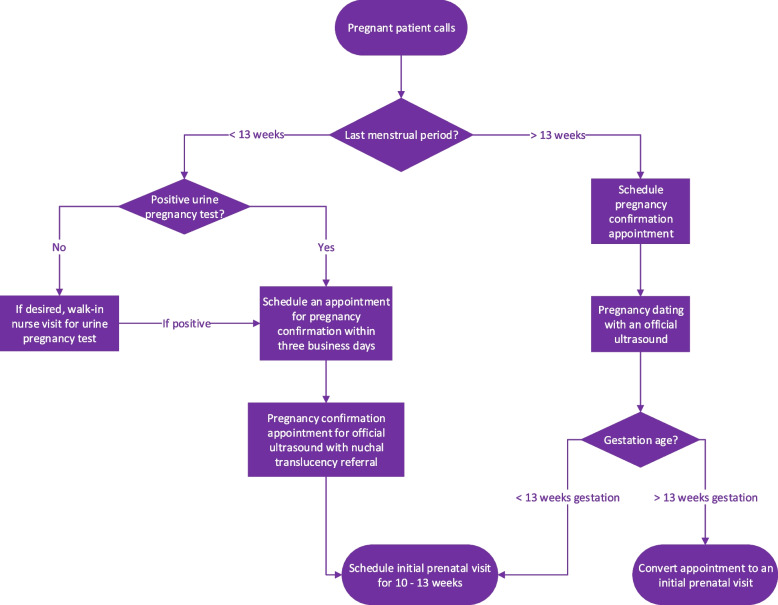
Entry to prenatal care at FHC at NYU Langone

The following diagram (Fig. 1) details the clinic workflow in place for patients to enter prenatal care.

## Methods

### Chart review and thematic analysis

A chart review was conducted on the 200 obstetrics patients that initiated prenatal care during their second or third trimester – i.e., after 13 weeks and 6 days of gestation – at FHC between August, 2021 and July, 2022. These patients, as well as additional data pertaining to relevant demographics, were identified using a customized report developed by Azara Healthcare for NYU Langone FHC’s annual quality reporting. This report leveraged structured data in Epic EMR and used custom logic to identify patients who were pregnant and their respective trimesters of entry.

The chart review was conducted by one reviewer (VNH). For each patient identified as entering prenatal care > 14 weeks, VNH accessed the patient’s medical record number in Epic EMR to open the patient’s chart. VNH identified the pertinent notes, particularly the provider notes from the patient’s pregnancy confirmation and initial prenatal visit (IPV). From these notes, VNH collected and compiled relevant data points, including age at IPV, date of positive pregnancy test (+ UPT) or confirmation bedside ultrasound (BSUS), date of IPV, weeks at entry to care, and number of no show visits, into a Microsoft Excel File.

For each patient, primary and secondary reasons for delay in prenatal care were selected from a list curated by FHC OB providers, based on common reasons for delay anecdotally expressed by patients. VNH also observed additional details or further explanations of the delay in the free text of the patient’s medical record and captured this data in a Comments section. These Comments included free text data fields from visits to the Emergency Department prior to the IPV and provided nuanced reasons for delay. Thus, these Comments served as the basis for the thematic analysis. Using a socio-ecological framework, the thematic analysis was conducted with emergent coding and done by assigning one or two themes to each patient based on the Comments for each patient.

### Quantitative analysis

To facilitate interpretation and analysis of these data, a reference population of all prenatal patients who entered prenatal care at the FHC in their first trimester during the same measurement period was then drawn directly from Epic databases by the FHC’s data team (*n* = 727). These patients were representative of the prenatal patient population at the FHC, and their inclusion facilitated two methods of quantitative analysis, detailed below.

First, we are simply interested in the dimensions along which patients who entered care late differ from those who entered care during their first trimester. To this end, the statistical significance of differences between the two populations regarding patient demographics (race, ethnicity, primary language, and age), engagement (number of FHC visits prior to their IPV), and pregnancy metrics (including gestational age at IPV) were evaluated using non-parametric tests. Specifically, the significance of differences in the central tendencies of continuous variables were evaluated using the Mann–Whitney U test, while differences in proportions were evaluated using the Chi-Squared test. To account for the possibility of heterogeneity across demographic groups, we also evaluated the differences in engagement and pregnancy metrics by race and ethnicity, and by primary language (Table 1). Lastly, we similarly assessed the prevalence of each ‘Theme’ broken down by race and ethnicity, primary language, and by age cohort (Tables 3 and 4).

**Table 1:** Patient descriptive statistics

	**Delayed prenatal care**	**Timely prenatal care**
*N*	169	727
*% Black (non-Hispanic)*	11.20	9.10
*% Hispanic*	80.00	79.10
*% Asian (non-Hispanic)*	1.80	4.10
*% White (non-Hispanic)*	2.40	4.40
*% Other race/ethnicity*	4.60	3.30
*% Spanish speakers*	64.00*	54.40
*% English speakers*	33.10*	41.80
*% Arabic speakers*	1.78	0.80
*% Other language speakers*	1.20	0.14
*Mean age at IPV*	27.80***	30.30
*Max age at IPV*	43.00	47.09
*Min age at IPV*	15.00	14.50
*Mean weeks at IPV*	19.10***	9.90
*Max weeks at IPV*	40.00	13.00
*Min weeks at IPV*	14.00	0.30
*Mean days to IPV*	35.70***	7.40
*Max days to IPV*	141.00	62.70
*Min days to IPV*	0.00	0.00
*Max visits at IPV*	75.00	474.00
*Min visits at IPV*	1.00	1.00
*Mean visits at IPV*	11.57***	21.70

The difference in the central tendencies of continuous variables compared between delayed patients and timely patients was evaluated using the Mann–Whitney U test. Differences in proportions were evaluated using a Chi-Squared test. We specify statistical significance to the 10% (.), 5%(*), 1%(**), and .1%(***) levels

In addition to the reasons and themes underlying delayed entry into prenatal care, the determinants associated with this delay are also of interest as they may reveal which patients are the most at risk of delayed entry, going forward. To evaluate the outcome of interest (i.e. whether or not a patient entered into care within their second or third trimester), we implemented a logistic regression model. In addition to the inclusion of patient age, race and ethnicity (adhering to HRSA’s definitions as they are standard practice at FHC), and primary language as covariates, we also regressed upon each patient’s prior engagement with FHC (proxied by the number of visits at FHC prior to their IPV), their expected delivery date (which captured the effect of any time trends), and the site at which they received care. This last covariate carries particular importance as each site varies not only in regard to patient demographics but also capacity and infrastructure.

### Social determinants of health—screening

While these covariates may be sufficient to reasonably model the risk of delayed entry, FHC has also incorporated systematic monitoring of the prevalence of social determinants of health (SDOHs) across its population, such that the standard practice is to screen all new patients for these SDOHs (see Appendix, Table 12 for full list). However, as the true screening rate varies by site, we must account for the patients for whom these data are missing in order to incorporate these SDOHs into the model (see Appendix, Table 9 for data missingness). We first assessed whether the patients who had not been screened were not screened effectively at random, implementing a preliminary logistic regression with screening status as the binary outcome variable and regressing on patient demographics, estimated delivery date, and site. The results of this regression, found in Table 10 in the Appendix, indicate that the likelihood of being screened is not related to patient demographics but is strongly related to the site at which the patient received care and whether the patient’s IPV was their first FHC visit. The likelihood is similarly increasing in regard to prior FHC engagement, as we would expect.

### Multiple imputation by chained equations

Having determined that these data are not missing completely at random, such that censoring these data would likely introduce bias into our estimates, we implemented multiple imputation by chained equations (MICE) in R using the ‘mice’ package developed by Van Buuren and Groothuis-Oudshoorn [21]. MICE has seen broad applications in similar settings across the public health and epidemiological literature, and overcomes the barrier of missing data by essentially imputing multiple potential iterations of these data, regressing on each, and pooling the coefficients [22, 23]. The potential for introducing bias via these methods is reduced when including the maximum number of covariates possible [23]. The ‘mice’ package specifically implements conditional multiple imputation wherein the underlying distribution of each variable suffering from missingness is modeled *conditional upon* the other variables present in the data. Electing to impute five distinct iterations of these data, we successfully included binary indicators reflecting whether or not a patient had experienced a given SDOH into the logistic regression model; due to collinearity, some of the SDOHs found in the full list in the Appendix were excluded.

## Results

Out of 200 patients, three were found to be duplicates and a further 28 patients had simply been mislabeled as entering in their second or third trimester. Thus, these 31 patients were removed from further analysis, resulting in a population of 169 delayed patients. The distribution of the age of OB patients that experienced delayed initiation of prenatal care was bimodal and non-normal, with a range of 15 – 43 years old and a mean of 27.8 years old.

The gestational age at entry of care ranged between 14 to 40 weeks, with a mean of 19 weeks. Among OB patients receiving prenatal care, no show visits, where the patient did not show up to an appointment, varied between zero to eight visits, with the median number being two. The One Key Question (pre-conception counseling) was utilized in 11.0% of this patient population. Sixty-one percent of these 169 patients did not receive primary care or have an annual GYN appointment in the year before their IPV.

As shown in Table 1 on patient descriptive statistics, mean testing confirms that the delayed population was significantly younger than those who entered prenatal care in the first trimester, for whom the mean age was just over 30 years old. Conversely, the population of delayed patients did not differ significantly from their timely-entry peers with regard to race and ethnicity. Those in the delayed group were 80.0% Hispanic/Latino, 11.2% African American (non-Hispanic), 1.8% Asian (non-Hispanic), and 2.4% White (non-Hispanic), with a further 4.6% belonging to an additional racial or ethnic group. That said, delayed patients were significantly more likely to select Spanish as their primary language and, conversely, were less likely to be primary English speakers.

With regard to FHC engagement and pregnancy characteristics, patients who experienced delayed entry to care were significantly less likely to have received care at FHC prior to their initial prenatal appointment (11.6 prior appointments compared to the almost 17 prior appointments of their non-delayed counterparts). Crucially, delayed patients experienced a delay between pregnancy confirmation and initiation of prenatal care almost five times the length of the delay experienced by patients who entered care on time.

### Reasons for delay

**Fig. 2:**
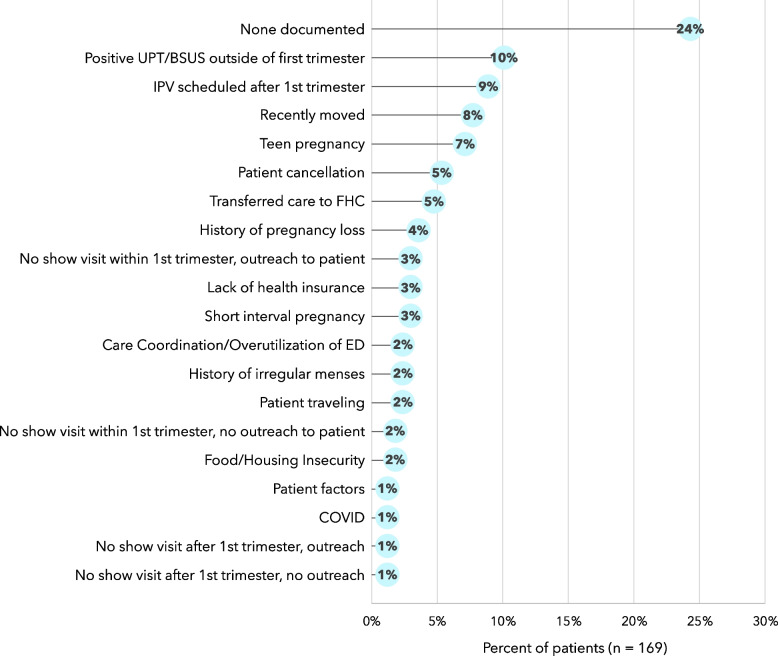
Primary reasons for delay in a chart review of 169 patients that initiated prenatal care after their first trimester at a federally qualified health center in Brooklyn, NY

As depicted in Fig. 2, 24% of patients did not have a reason documented in their medical record for their late entry to care. The chart review identified 10.0% of patients that had late entry due to a + UPT/BSUS after the first trimester. These patients initially thought they were earlier in their pregnancy and thus, came in for their IPV later. Nine percent of patients had difficulty scheduling their IPV during their first trimester. Eight percent of patients recently moved to the Brooklyn area after arriving from other parts of the country or from outside of the United States. Teenage pregnancy accounted for 7.0% of this patient population. See Appendix for full list of primary reasons for delay (Table 6) and secondary reasons for delay (Table 7).

Additional phone follow-up to the 40 patients without a documented reason for delay was done by an FHC community health worker. This outreach revealed that nine did not know they were pregnant until later in their pregnancy, one had insurance issues, and another had difficulty scheduling. The remaining patients were unable to be reached because they did not pick up, provided incorrect contact information, or had disconnected service.

**Fig. 3:**
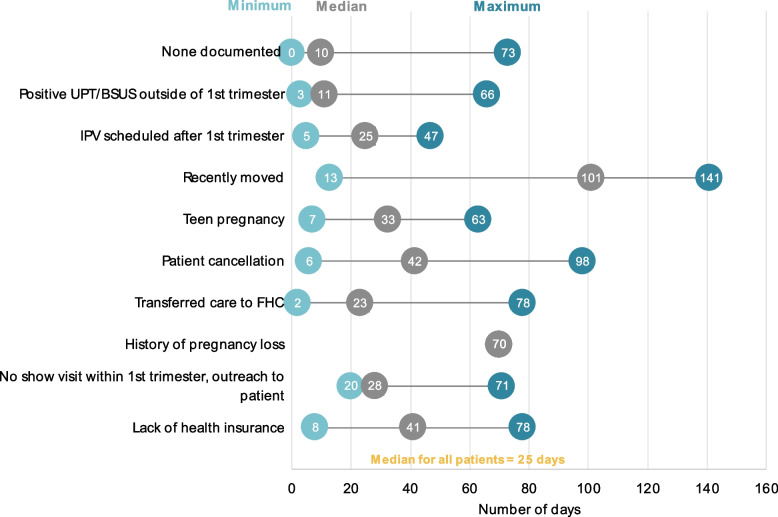
The minimum, median, and maximum number of days between a patient's positive pregnancy confirmation and initiation of prenatal care among the top eleven primary reasons for delay (*n* = 169)

As illustrated in Fig. 3, the median number of days between a positive pregnancy confirmation and their IPV for all 197 patients was 25 days. Some patients with no reason for delay documented had their pregnancy confirmation appointments immediately converted to their first prenatal visit, thus entering prenatal care once learning they were pregnant (minimum = 0). Patients who recently moved to the Brooklyn area had the largest range in days between pregnancy confirmation and entering prenatal care, with the median number of days being 101. Patients who canceled their IPV had a median number of 42 days between their pregnancy confirmation and entering prenatal care.

### Themes identified

As shown in Table 2, the most common themes identified in the chart review (*n* = 155) were the patient being in transition, the pregnancy being unplanned, and issues with linkage to care, including no shows or patient cancellations. As in most major cities, NYC has a culture of transience, which lends to difficulty in establishing health care. This was a theme seen in 33 patients’ charts (21.2%). Twenty-five patients reported that their pregnancy was unplanned, often either the result of it being a teen pregnancy or a short interval pregnancy from lack of contraceptive use after their last pregnancy (16.8%). Linkage to care was a common issue, with 24 patients either canceling or not showing up for their IPV and 15 patients scheduling their IPVs after their first trimester (15.5%). Fifteen patients did not know they were pregnant due to having a history of irregular menses (9.7%). Additional themes identified are reported in Table 2.

**Table 2:** Themes, definitions, and corresponding quotes identified in chart review (*n* = 155)

Theme	Definition	Count	%	Quote(s)
Patient in transition	The patient was recently traveling for an extended period of time, recently relocated, transferred her care to FHCs, or transferred her care elsewhere	33	21.2	• “Patient was in Uzbekistan from August to January." • "Patient recently moved from Guatemala in 01/22. No prenatal care in Guatemala." • “Patient transferred her care from Gouverneur Women's Health Services (which began at 8 weeks) to FHCs (at 22 weeks).” • “Patient appears to not have continued PNV with FHCs—on 2/7/22, she asked for a summary of her initial visit and then no showed or canceled subsequent appointments via the reminder.”
Unplanned pregnancy	The patient had an unplanned pregnancy as the result of a short interval pregnancy or teen pregnancy	26	16.8	• “This is an unplanned, but desired pregnancy. Patient had a c-section for the birth of her last child in 5/2021 (< 8 months ago). At her postpartum visit, she stated that she would start OCPs for birth control.” • “Patient presented to the ED on 1/13/22 stating that she had two positive home pregnancy tests and wanted to initiate prenatal care. This is an unplanned but desired pregnancy.” • “Denied sexual activity during HEADSS assessment at PCP visit prior to IPV."
Linkage to care: no show/patient cancellation	Patient did not show up for her scheduled appointment or canceled her scheduled appointment	24	15.5	• "Patient had two no show appointments and canceled an appointment in the two months prior to her IPV."
Didn't know	Patient reports a history of irregular menstrual cycles or that she did not know she was pregnant	15	9.7	• "Patient has a history of irregular menses that are late by 2–3 months." • “At IPV, the patient reported her LMP was unknown. Patient was unaware that she is pregnant.”
Linkage to care: scheduling	Patient had a pregnancy confirmation at FHCs or the ED but was not scheduled for an appointment before the end of her first trimester	15	9.7	• "Patient was found to be pregnant at 9 weeks, but didn't have an IPV scheduled until 14 weeks."
Social determinants of health	The patient experienced food/housing insecurity, difficulty obtaining health insurance, or health literacy issues, which prevented her from seeking care sooner	15	9.7	• “Patient referred to social work for the following: Lack of adequate food; Inadequate housing; Unavailability and inaccessibility of health-care facilities; Insufficient social insurance or welfare support; Other problems related to housing and economic circumstances.” • "Per ED note, she was supposed to follow up with OB/GYN for prenatal care; however, states she did not have insurance and never called to make an appointment." • "Difficulty navigating the healthcare system—Did not complete blood work or anatomy scan prior to second prenatal visit because she was confused about where to go." • "Patient didn't know she needed to come earlier to initiate prenatal care.”
Pregnancy loss	The patient has a history of spontaneous abortion in previous pregnancies or lost her current pregnancy	10	6.5	• “Patient has a history of 3 c-sections and recurrent miscarriages in the first trimester.” • “At IPV, she endorsed vaginal spotting for 3 weeks. Prior to spotting, she had some vaginal bleeding one month ago and went to ED out of state. Denied abnormal findings at ED visit. At IPV, no fetus or FHR was found on US. Patient was sent to ED for evaluation of miscarriage."
Childcare	The patient has multiple children at home and as a result, was unable to attend prenatal care earlier	6	3.8	• "She has not received prenatal care because she states that she was busy caring for the [3] children at home. She reports that now her home life is stable and she will be able to present for her visit."
Ambivalence	The patient was unsure about continuing the pregnancy	4	2.6	• "At IPV, she stated that she came late to start prenatal care because she was thinking about not to keeping the pregnancy."
Overutilization of ED services	The patient utilized the emergency department for primary care instead of establishing early prenatal care	4	2.6	• "In the ED, patient had a + UPT on 11/30/21 and then a missed abortion on 12/6/21. Returned to the ED on 1/22/22 for evaluation of headache; had BSUS which revealed IUP at 7 weeks. Went back to ED on 3/23/22 at 14 weeks gestation, on 4/18/22 and 4/22/22 at 19 weeks."
Patient factors	The patient has a history of mental health illness that impacted her ability to seek care	3	1.9	• "Patient has a history of depression and alcohol use during pregnancy… Previous pregnancy was late to care at 34 weeks."
*TOTAL*		**155**	100	

Among Hispanic patients in this subset (*n* = 135), the most common themes noted were none documented (19.3%), no show (14.8%), and in transition (12.6%). In patients that preferred Spanish, the most common themes were none documented (22.2%) and no show (14.8%). By age, 18-year-olds and under *(n* = 12) were most likely to have unplanned pregnancies (58.3%). Nineteen to 24-year-olds (*n* = 42) were most likely to be in transition (28.6%). Patients between 25 – 30 years old (*n* = 64) more often had no reason documented (27.7%). Finally, patients that were older than 30 years old (*n* = 50) were most likely to no show (26.0%). See Tables 3 and 4 for additional breakdowns of themes by race, language, and age.

**Table 3:** Theme descriptive table by race and primary language

			**Race**			**Language**			
	**Hispanic** (*n* = 135)	**Black **(*n* = 19)	**Asian **(*n* = 3)	**White **(*n* = 4)	**Other Race **(*n* = 8)	**Spanish **(*n* = 108)	**English **(*n* = 56)	**Arabic **(*n* = 3)	**Other **(*n* = 2)
***Ambivalence %***	2.2	5.3	0.0	0.0	0.0	0.0	7.1	0.0	0.0
***Loss of Pregnancy %***	5.2	0.0	0.0	0.0	12.5	3.7	7.1	0.0	0.0
***SDOH %***	8.9	5.3	0.0	0.0	12.5	11.1	3.6	0.0	0.0
***No Show %***	14.8	26.3	0.0	0.0	12.5	14.8	17.9	0.0	0.0
***Unplanned %***	11.9	15.8	0.0	0.0	0.0	11.1	12.5	0.0	0.0
***Scheduling %***	10.4	0.0	0.0	0.0	0.0	10.2	5.4	0.0	0.0
***In Transition %***	12.6	26.3	66.7	25.0	37.5	10.2	25.0	66.7	50.0
***Didn't Know %***	11.1	0.0	33.3	0.0	0.0	12.0	5.4	0.0	0.0
***Childcare %***	3.7	0.0	0.0	0.0	0.0	3.7	1.8	0.0	0.0
***Patient Factors %***	5.2	5.3	0.0	25.0	0.0	5.6	3.6	0.0	50.0
***Overutilization %***	3.0	0.0	0.0	0.0	0.0	2.8	1.8	0.0	0.0
***None %***	19.3	26.3	0.0	50.0	25.0	22.2	17.9	33.3	0.0

**Table 4:** Theme descriptive table by age cohort

	***18 or younger ****(n* = *12)*	***19–24 ****(n* = *42)*	***25–30 ****(n* = *65)*	***Older than 30 ****(n* = *50)*
*Ambivalence %*	16.7	2.4	1.5	0.0
*Loss of Pregnancy %*	8.3	4.8	6.2	2.0
*SDOH %*	8.3	9.5	12.3	2.0
*No Show %*	8.3	4.8	15.4	26.0
*Unplanned %*	58.3	14.3	6.2	4.0
*Scheduling %*	0.0	4.8	7.7	14.0
*In Transition %*	8.3	28.6	12.3	14.0
*Didn't Know %*	8.3	7.1	7.7	14.0
*Childcare %*	0.0	0.0	3.1	6.0
*Patient Factors %*	8.3	2.3	6.2	6.0
*Overutilization %*	0.0	4.8	1.5	2.0
*None %*	0.0	21.4	27.7	16.0

### Determinants of delay

Now turning to the identification of the primary determinants of delayed entry to care, Table 5 holds the results from the logistic regression of delayed status on patient demographic and FHC engagement covariates. Separate specifications of the model were run for the exclusion and inclusion of SDOH data as regressors, reflected in columns A and B, respectively.

**Table 5:** Logistic regression of delayed status on patient characteristics

	**Without SDOHS (A)**	**With SDOHS (B)**
*Age*	0.925*** (0.016)	0.923*** (0.018)
*Race: Other*	2.191 (0.735)	2.374 (0.754)
*Race: Black*	4.826* (0.737)	4.771* (0.761)
*Race: Asian*	1.454 (0.887)	1.575 (0.914)
*Race: Hispanic/Latino*	1.203 (0.613)	1.233 (0.631)
*Language: Spanish*	2.368 (0.864)	2.415 (0.888)
*Language: English*	1.323 (0.840)	1.342 (0.863)
*Language: Arabic*	3.277 (1.124)	3.268 (1.151)
*FHC Site B*	10.358• (1.239)	7.005 (1.438)
*Estimated Delivery Date*	0.993*** (0.001)	0.993*** (0.001)
*Visits prior to IPV*	0.974*** (0.007)	0.973*** (0.007)
*SDOH: Food Insecurity*		0.307 (1.858)
*SDOH: Transportation*		0.811 (0.648)
*SDOH: Utilities*		1.368 (0.665)
*SDOH: Education*		3.281 (1.190)
*SDOH: Physical Activity*		0.770 (0.280)
*SDOH: Stress*		0.999 (0.632)

We implemented a logistic regression model to estimate the marginal effects of key demographics determinants on whether a patient experienced delayed entry to care. Odds ratios are presented with standard errors in parentheses. We specify statistical significance to the 10% (.), 5%(*), 1%(**), and 0.1%(***) levels, with α = .05. Due to lack of statistical significance, the odds ratios associated with four of the five remaining FHC sites are not presented here but can be found in the full table in the Appendix

Across each specification of the model, the odds of a patient experiencing delayed entry to care decreases with age by approximately 7.5% per additional year; this effect is statistically significant to the 0.1% level. All else equal, older patients exhibit a lower likelihood of delayed entry to care than their younger peers. Similarly, the likelihood of delayed entry is decreasing in estimated delivery date (which proxies for the relative timing of prenatal care within the measurement period) and prior engagement at FHC. These indicate that the frequency of delayed entry trended down over time and that patients that were seen more often at FHC were also more likely to receive prenatal care on time.

In contrast with the conclusions drawn from Table 1. Patient Descriptive Statistics, race, ethnicity, and language did not contribute significantly to delayed status, with the exception of a patient being black for whom, on average delayed entry was nearly five times more likely. Lastly, though not significant at the 5% level, receiving prenatal care at FHC Site B is associated with a significant increase in the likelihood of delayed entry compared to the control. However, as the statistical significance of this effect disappears when including the SDOHs in the full sample specification, we cannot confidently attribute this effect to the site, itself.

## Discussion

This was a mixed-methods study conducted to identify patient-perceived barriers to early entry of prenatal care and patient characteristics that are more at-risk for delayed entry among a vulnerable patient population serviced by an FQHC. As FHC prepares to launch initiatives around improving maternal and child health, this study provides a baseline to measure future progress.

### Reasons for delay and themes identified

Among the 169 patients that initiated prenatal care in their second or third trimester between August 2021 – July 2022, 24% lacked documentation regarding a reason for delay and 9% had difficulty scheduling an initial prenatal visit within their first trimester. Thus, this chart review highlighted an area of improvement for FHC. However, this study also underscores the importance of FQHCs as safety net institutions in urban settings, given than 10% did not learn of their pregnancy until after their first trimester, 8% recently moved to Brooklyn from outside of NYC or the USA, and 7% were teenage pregnancies. This is further reinforced with the themes identified (*n* = 155) of patients being in transition (21%) and the pregnancy being unplanned (17%). For issues with linkage to care (15%), including no shows or patient cancellations, closer follow-up of patients with a recent pregnancy confirmation is warranted.

### Determinants of delayed entry

Further quantitative analysis revealed that these patients differed significantly from those FHC patients who entered into prenatal care within their first trimester. Delayed patients were more likely to be Spanish speakers, to be young, and to experience relatively long delays between pregnancy confirmation and entry into care; this latter finding is consistent with the results of the thematic analysis discussed above. Moreover, irrespective of the model used, patient age at the initial prenatal visit remained the primary determinant of delayed entry. Essentially, patients that are older are more likely to be compliant to first trimester entry into care, and those younger may be at risk of late entry, which is consistent with prior literature. Even when controlling for SDOHs, patient age holds a great deal of predictive power in this setting and may warrant greater attention placed upon the themes salient to the two youngest cohorts. Of note, controlling for SDOHs minimally affected the results, with the exception of the aforementioned effect associated with Site B. This indicates that variation associated with SDOHs was largely driven by other covariates, particularly the site at which patients received care. Interestingly, as Table 8 in the Appendix demonstrates that the proportion of prenatal patients experiencing delay was generally lower in the smaller sites and given that the largest site (Site A) was not a significant determinant of delayed status, site capacity and infrastructure do not seem to be determinants of delayed status in this setting. Rather, patient engagement with the FHC prior to their IPV and patient age account for much of the variation in timing of entry.

### Future directions

FHC continues to strive for excellence in early initiation of prenatal care in their OB patients. In response to findings from the chart review, if prenatal patients are flagged as late to care at their IPV, providers are now required to document the reason. Consistent documentation in the Epic EMR will be supported with the introduction of a smart data element that can be easily populated into IPV notes. This data element will allow prenatal providers to select the reason for delay from a dropdown list.

Furthermore, this study has prompted FHC leadership to revisit the availability of initial prenatal visit appointments and re-emphasize the workflow of converting pregnancy confirmation appointments to initial prenatal visits if the dating ultrasound reveals a gestation age over 13 weeks. Efforts to address no shows and patient cancellations in the first trimester should also be documented by front desk staff or community health workers.

As seen in other studies on barriers to prenatal care, patient age was the greatest determinant of timely entry to prenatal care in our patient population. Thus, increasing priority should be placed on tailoring adolescent health interventions in collaboration with FHC’s school health clinics. As FHC prepares to launch additional maternal and child health initiatives, the sum of these findings support designating community health workers for targeted patient follow-up, particularly for those with unplanned pregnancies and patients in transition, including those who recently moved to the area.

While FHCs is proud that 50% of these patients entered into care ≤ 25 days after their pregnancy confirmation, collaborative efforts can be made to decrease this gap so that 75% enter into prenatal care in under a month after pregnancy confirmation.

### Strengths and limitations

A strength of our study is that it was conducted in a medically vulnerable patient population. These findings can be utilized to identify patient characteristics that may warrant closer follow-up and longer appointments slots to ensure adequate time for patient education. A limitation of our study, and all retrospective studies, is that of the patient history. It is challenging to discern how accurate the transfer of information was from the patient to the provider to the medical record. Each patient had a varying level of understanding to the question that was inquired of them – “why were you late to starting prenatal care?”. Based on their response, the provider may have interpreted it differently than intended or added context to their explanation while documenting it in the record. Another limitation includes the large proportion of patients that lacked documentation regarding a reason for their delay. Without documentation, we are confined to analyzing quantitative measures, such as patient demographics or the SDOH screener, to deduce what caused the delay.

## Conclusions

This study reflects the importance of involving patient and stakeholders in building an office practice model to better improve timely prenatal care. Future initiatives include the introduction of a smart data element to encourage consistent documentation of the reason for delay. Additionally, we aim to dedicate community health workers for targeting outreach and follow-up after delayed prenatal patients have no show appointments or cancel without rescheduling. We plan for evaluation of future quality improvement initiatives to determine their impact on reducing delayed initiation of prenatal care and mapping that to improved quality of care and positive maternal and infant outcomes at delivery.

### Supplementary Information

Supplementary Material 1.

## Data Availability

The datasets used and analyzed during the current study are available from the corresponding author on reasonable request.

## Abbreviations

BSUS: Bedside ultrasound
ED: Emergency department
EMR: Electronic medical record
FHC: Family Health Centers
FHR: Fetal heart rate
FQHC: Federally qualified health center
GYN: Gynecology
HEADSS: Home, education, activities/employment, drugs, suicidality, and sex
HRSA: Health resources and services administration
IMR: Infant mortality rate
IPV: Initial prenatal visit
LMP: Last menstrual period
MICE: Multiple imputation by chained equations
NYU: New York University
OB: Obstetric
PNC: Prenatal care
SDOH: Social determinants of health
UPT: Urine pregnancy test
