# Supplementary material for: Patient-perceived barriers to early initiation of prenatal care at a large, urban federally qualified health center: a mixed-methods study
Source: BMC Pregnancy Childbirth. 2024 Jun 21;24:436. doi: 10.1186/s12884-024-06630-9 (PMC11193180; doi:10.1186/s12884-024-06630-9)
Supplement: Supplementary file 1 — Supplementary Material 1. [file 12884_2024_6630_MOESM1_ESM.docx]

**APPENDIX**

**Table 6.** **The Primary Reasons for Delay in Initiation of Prenatal Care (N = 169)**

| **Primary Reasons for Delay** | **# of patients** | **%** |
| --- | --- | --- |
| None documented | 41 | 21% |
| Positive UPT/BSUS outside of first trimester | 17 | 9% |
| IPV scheduled after 1st trimester | 15 | 8% |
| Recently moved | 13 | 7% |
| Teen pregnancy | 12 | 6% |
| Patient cancellation | 9 | 5% |
| Transferred care to FHCs | 8 | 4% |
| History of pregnancy loss | 6 | 3% |
| No show visit within 1st trimester, outreach to patient | 5 | 3% |
| Lack of health insurance | 5 | 3% |
| Short interval pregnancy | 5 | 3% |
| Care Coordination/Overutilization of ED | 4 | 2% |
| History of irregular menses | 4 | 2% |
| Patient traveling | 4 | 2% |
| No show visit within 1st trimester, no outreach to patient | 3 | 2% |
| Food/Housing Insecurity | 3 | 2% |
| Patient factors | 2 | 1% |
| COVID | 2 | 1% |
| No show visit after 1st trimester, outreach | 2 | 1% |
| No show visit after 1st trimester, no outreach | 2 | 1% |
| Patient undecided on pregnancy | 1 | 1% |
| Transferred care to elsewhere | 1 | 1% |
| Unaware of the importance of prenatal care | 1 | 1% |
| Immigration | 1 | 1% |
| Health Literacy | 1 | 1% |
| Childcare issues | 1 | 1% |
| Total | 169 | 100% |
|  |  |  |

**Table 6.** The top three primary reasons for delay in initiation of prenatal care were none documented, positive UPT/BSUS outside of first trimester, and IPV scheduled after first trimester.

**Table 7. The Secondary Reasons for Delay in Initiation of Prenatal Care (N = 29)**

| **Secondary Reasons for Delay** | **# of patients** | **%** |
| --- | --- | --- |
| History of irregular menses | 5 | 18% |
| Childcare issues | 3 | 11% |
| Lack of health insurance | 3 | 11% |
| Patient undecided on pregnancy | 3 | 11% |
| History of pregnancy loss | 2 | 7% |
| Patient cancellation | 2 | 7% |
| Short interval pregnancy | 2 | 7% |
| Recently moved | 1 | 4% |
| Transferred care to FHCs | 1 | 4% |
| No show visit within 1st trimester, outreach to patient | 1 | 4% |
| Care Coordination/Overutilization of ED | 1 | 4% |
| Patient factors | 1 | 4% |
| COVID | 1 | 4% |
| Transferred care to elsewhere | 1 | 4% |
| Unplanned pregnancy | 1 | 4% |
| Transportation issues | 1 | 4% |
| **Total** | **29** | **100%** |

**Table 7.** The top secondary reason for delay in initiation of prenatal care was having a history of irregular menses.

**Table 8. Site Breakdown of Delayed Patients**

| FHC Site | Delayed | Reference | Proportion Delayed |
| --- | --- | --- | --- |
| Site A | 134 | 518 | 20.6% |
| Site B | 5 | 15 | 25.0% |
| Site C | 5 | 44 | 10.2% |
| Site D | 11 | 65 | 14.5% |
| Site E | 10 | 55 | 15.4% |
| Site F | 1 | 30 | 3.2% |

**Table 9. Missingness Table**

| Covariate | Percent Missing |
| --- | --- |
| MRN | 0.0% |
| Age At IPV | 0.0% |
| Race/Ethnicity | 0.0% |
| Preferred Language | 0.0% |
| FHC Site | 0.3% |
| Gestational Age At IPV | 0.1% |
| Number Of FHC Visits Prior To IPV | 0.3% |
| Working Estimated Delivery Date | 0.1% |
| SDOH: Food Insecurity | 29.4% |
| SDOH: Transportation Barrier | 29.4% |
| SDOH: Utilities | 29.4% |
| SDOH: Education | 29.4% |
| SDOH: Physical Activity | 29.4% |
| SDOH: Stress | 29.4% |

**Table 10. Logistic Regression of whether a patient received an SDOH screener on patient characteristics**

| Age at IPV | 0.966•  (0.020) |
| --- | --- |
| Race: Other | 4.190  (1.110) |
| Race: Black | 0.989  (0.760) |
| Race: Asian | 3.853  (0.857) |
| Race: Hispanic | 0.793  (0.564) |
| Language: Spanish | 2.923  (0.802) |
| Language: English | 1.350  (0.759) |
| Language: Arabic | 0.702  (1.457) |
| FHC Site A | 84.344***  (0.685) |
| Expected Delivery Date | 0.995***  (0.001) |
| FHC Visits prior to IPV | 1.014**  (0.005) |
| IPV was Patient’s First Visit | 0.267**  (0.488) |

**Table 10.** We implemented a logistic regression model to determine whether the SDOH screening status of the patient population varied with any of the covariates of interest and were thus not missing completely at random (MCAR). Odds ratios are presented with standard errors in parentheses. We specify statistical significance to the 10% (.), 5%(*), 1%(**), and .1%(***) levels, with α = .05.

**Table 11. Full Main Regression Table**

|  | Without SDOHs | With SDOHs |
| --- | --- | --- |
| **Age** | 0.925***  (0.016) | 0.923***  (0.018) |
| **Race: Other** | 2.191  (0.735) | 2.374  (0.754) |
| **Race: Black** | 4.826*  (0.737) | 4.771*  (0.761) |
| **Race: Asian** | 1.454  (0.887) | 1.575  (0.914) |
| **Race: Hispanic/Latino** | 1.203  (0.613) | 1.233  (0.631) |
| **Language: Spanish** | 2.368  (0.864) | 2.415  (0.888) |
| **Language: English** | 1.323  (0.840) | 1.342  (0.863) |
| **Language: Arabic** | 3.277  (1.124) | 3.268  (1.151) |
| **FHC Site A** | 4.109  (1.094) | 5.446  (1.615) |
| **FHC Site B** | 10.358•  (1.239) | 7.005•  (1.438) |
| **FHC Site C** | 1.989  (1.195) | 2.094  (1.245) |
| **FHC Site D** | 1.331  (1.206) | 1.486  (1.327) |
| **FHC Site E** | 4.630  (1.148) | 5.650  (1.218) |
| **Estimated Delivery Date** | 0.993***  (0.001) | 0.993***  (0.001) |
| **Visits prior to IPV** | 0.974***  (0.007) | 0.973***  (0.007) |
| **SDOH: Food Insecurity** |  | 0.307  (1.858) |
| **SDOH: Transportation** |  | 0.811  (0.648) |
| **SDOH: Utilities** |  | 1.368  (0.665) |
| **SDOH: Education** |  | 3.281  (1.190) |
| **SDOH: Physical Activity** |  | 0.770  (0.280) |
| **SDOH: Stress** |  | 0.999  (0.632) |

**Table 11.** We implemented a logistic regression model to estimate the marginal effects of key demographics determinants on whether a patient experienced delayed entry to care. Odds ratios are presented with standard errors in parentheses. We specify statistical significance to the 10% (.), 5%(*), 1%(**), and .1%(***) levels, with α = .05.

**Table 12. Full List of SDOHs**

| Food Insecurity |
| --- |
| Housing Insecurity |
| Financial Strain |
| Transportation Barriers |
| Utilities |
| Safety |
| Employment |
| Education |
| Physical Activity |
| Mental Health |
| Stress |
| Social Isolation |
| Health Literacy |
| Legal |
| Childcare |
